# Association between dietary antioxidant levels and chronic obstructive pulmonary disease: a mediation analysis of inflammatory factors

**DOI:** 10.3389/fimmu.2023.1310399

**Published:** 2024-01-08

**Authors:** Ziyi Liu, Jiyuan Li, Tuotuo Chen, Xue Zhao, Qing Chen, Lihua Xiao, Zhenyu Peng, Hongliang Zhang

**Affiliations:** ^1^ Department of Emergency Medicine, The Second Xiangya Hospital, Central South University, Changsha, China; ^2^ Emergency and Difficult Diseases Institute of Central South University, Changsha, China; ^3^ Department of Kidney Transplantation, Center of Organ Transplantation, The Second Xiangya Hospital, Central South University, Changsha, China; ^4^ College of Medicine, Hunan Normal University, Changsha, China

**Keywords:** chronic obstructive pulmonary disease, antioxidant, diet, inflammatory factor, national health and nutrition examination survey

## Abstract

**Introduction:**

The development of chronic obstructive pulmonary disease (COPD) is strongly associated with oxidative stress, but it is unclear whether increasing dietary antioxidant intake reduces the risk of COPD. Therefore, this study assessed the association between antioxidant intake and COPD in US adults aged ≥ 40 years and further examined the correlation using the Composite Dietary Antioxidant Index (CDAI).

**Methods:**

The study included 8,257 US adults aged ≥ 40 years using data from the National Health and Nutrition Examination Survey (NHANES) for three cycles from 2007-2012. Multivariate logistic regression models were used to calculate the correlation between antioxidant intake and CDAI with COPD. Restricted cubic spline was further used to explore the exposure-response relationship. Mediation analysis was used to explore the role of inflammatory factors in the association between CDAI and COPD.

**Results:**

This study included 8257 participants (4111 women [weighted, 50.7%]; mean [SD] age, 58.8 [11.2] years). In a multivariable-adjusted model of single antioxidant intake, a linear downward association between carotenoid intake and the incidence of COPD (P for trend = 0.052; Pnon- linear = 0.961). In a multivariable adjusted model for CDAI, this association is similarly present (P for trend = 0.018; Pnon-linear = 0.360). Multiple linear regression modeling showed that leukocytes (P = 0.002), alkaline phosphatase (P< 0.001), and c-reactive protein (P< 0.001) were negatively associated with CDAI levels. Meanwhile, mediation analysis revealed that alkaline phosphatase and c-reactive protein partially influenced the association between CDAI and COPD prevalence, with mediation ratios of 6.4% (P< 0.01) and 4.68% (P = 0.04), respectively.

**Conclusion:**

The risk of COPD decreased with increased carotenoid intake and CDAI. In addition, CDAI has been found to be strongly associated with inflammatory factors and can reduce the incidence of COPD by mediating inflammatory factors.

## 1 Introduction

Chronic obstructive pulmonary disease (COPD) is characterized by restricted airflow and can lead to a progressive and irreversible decline in lung function. Studies have shown that COPD is the third leading cause of disability-adjusted life years, with a global prevalence of 10.3% among people aged 30-79 years (1, 2). Therefore, the disease burden of COPD (especially for elderly people) is significant and it is urgent to find primary prevention measures for COPD.

The potential role of dietary factors in COPD has been noted in some studies. It has been shown that a western diet high in red meat and saturated fat increases the risk of COPD, while a high intake of fruit, grains and vegetables reduces the risk (3, 4). Similarly, there are studies that point to pro-inflammatory diet (high dietary inflammation index scores) may increase the risk of COPD (5, 6). All these results support the promise of dietary factors as an important factor in the prevention of COPD.

Composite dietary antioxidant index (CDAI) is commonly used to reflect dietary antioxidant levels, with increasing antioxidant capacity as the index increases. This index is often used to explore the relationship between antioxidant diets and disease. Previous studies have shown that high CDAI reduces the risk of various cancers, and studies have also shown an L-shaped association between CDAI and all-cause mortality in chronic kidney disease (CKD) stages 1-2 (7–9).

Oxidative stress is important in the progression of COPD and exploring the relationship between COPD and antioxidant diets would provide assistance in the prevention or treatment of the disease. However, to our knowledge, there are no studies on the relationship between CDAI and COPD. Therefore, in this study of a representative US sample, we used 8257 participants aged ≥ 40 years from the national health and nutrition examination survey (NHANES) 2007-2012 database to explore the potential association of dietary antioxidant levels with COPD.

## 2 Materials and methods

### 2.1 Data source and study design

NHANES is a nationally representative study designed to collect information about the health and nutrition of the American population. Due to the refined sampling design of NHANES, the sample can be weighted to represent the entire US population. In this study, 24-hour dietary recall weights (WTDR2D) were selected according to National Center for Health Statistics (NCHS) guidelines, and WTDR2D/3 was used based on the cycle selected. Data from 3 cycles of NHANES 2007-2012 were used for this study. First, we excluded participants aged< 40 (N=18679) and pregnant (N=754). In addition, we excluded participants with weights missing or zero (N=2182). Finally, for data reliability, we excluded participants whose antioxidant intake range above mean ± 3 standard deviation (SD) or carotenoid intake is zero (The range of carotenoid intake is too large and needs to be taken as a logarithm) (N=570). Ultimately, 8257 participants were included in this study (No COPD diagnostic missing) (**Supplementary Figure S1**). The NHANES database is approved by the National Centre for Health Statistics and can be accessed directly by researchers who meet eligibility requirements. All participants in the database sign an informed consent form.

### 2.2 Measurement of dietary antioxidant intake

Data on the intake of each dietary antioxidant was assessed by two 24-hour recall surveys. The first 24h was recorded face-to-face at a mobile examination center and the next 24-hour was recorded by telephone 3-10 days later. Specific intakes of each nutrient were assessed through the American Dietetic Research Food and Nutrient Database. The average of the two 24-h intakes was taken as the daily dietary intake for this study.

The CDAI was calculated from the mean dietary intakes of vitamin A, vitamin C, vitamin E, zinc, selenium and carotenoids obtained from two 24-hour recalls. A standardization of each antioxidant (x_i_) was performed by subtracting the gender-specific mean ( μ _i_) and dividing by the gender-specific standard deviation (s_i_) (10). See the following formulas for details.


$$CDAI\;=\;\sum_{i=1}^{6}\frac{X_{i}-\mu_{i}}{s_{i}}$$

### 2.3 Dependent variables and covariates

COPD is defined as having one of the following: 1. FEV1/FVC< 0.7 after bronchodilators 2. Self-reported emphysema 3. Age > 40 years, history of chronic bronchitis or smoking, treatment with medication for COPD (including mast cell stabilizers, leukotriene modulators, inhaled corticosteroids, selective phosphodiesterase-4 inhibitors).

We used factors potentially associated with antioxidant intake, CDAI and COPD as covariates (including participants’ age, sex, race, education, marriage, poverty index, body mass index (BMI), drinking status, smoking status, and disease status). The poverty-to-income ratio (PIR) is divided into three levels, low income (PIR ≤ 1), middle income (1< PIR ≤ 3), and high income (PIR > 3). BMI was defined as weight/(height)^2^ kg/m^2^. Drinking status was categorized as never drinking, former drinking, mild to moderate drinking (defined as ≤ 2 drinks/day for females or ≤ 3 drinks/day for males or binge drinking 2-4 days/month, and heavy drinking (defined as ≥ 3 drinks/day for females or ≥ 4 drinks/day for males for binge drinking ≥ 5 days/month). Smoking status was categorized as never smoking (smoked< 100 cigarettes lifetime), former smoking (smoked > 100 cigarettes, now not smoking), and currently smoking (smoked > 100, now smoking sometime or daily). Hypertension was defined as a mean diastolic blood pressure ≥ 80 mmHg or a mean systolic blood pressure ≥ 130 mmHg or self-reported or use of anti-hypertensive medication. Participants were considered to have diabetes if their fasting blood glucose level was > 7 mmol/L or random blood glucose > 11 mmol/L or oral glucose tolerance test 2 hours level > 11.1mmol/L or glycosylated hemoglobin > 6.5% or self-reported or use of anti-diabetic medication. Hyperlipidemia was defined as TG ≥ 200 mg/dl or TC ≥ 200 mg/dl or LDL ≥ 130 mg/dl or HDL< 40 mg/dl (male), 50 mg/dl (female) or use of lipid-lowering medication. CKD was defined as an estimated glomerular filtration rate< 60 mL/min/1.73m^2^ or a urinary albumin/creatinine ratio ≥ 30 mg/g. Cardiovascular disease was defined as self-reported (including coronary heart disease, congenital heart failure, heart attack, stroke, angina).

### 2.4 Statistical analysis

Random forest interpolation refers to a method of estimation or prediction using the Random Forest algorithm, where multiple decision trees are constructed, each trained on a different subset of data. Together, these decision trees form a “forest” that is used to make predictions. It can be applied to nonparametric interpolation of mixed-type data, especially when there are intricate interactions (11). Therefore we apply the method to deal with missing data. Categorical variables were expressed as the number of people before weighting (weighted percentages). Chi-square tests were used to compare between-group differences in categorical variables. Daily dietary antioxidant intake (zinc, selenium, vitamin A, vitamin C, vitamin E, carotenoids) and CDAI were categorized into four groups (Q1, Q2, Q3 and Q4) based on quartile levels. Multivariate logistic regression analysis was used to explore the relationship between antioxidant intake and CDAI with the incidence of COPD (model1: unadjusted variables; model2: adjusted for: age, ethnicity, and sex; model3: adjusted for all potential covariates). Restricted cubic spline (RCS) curves are often used in statistical modeling to represent a nonlinear relationship between two variables. We further used the multivariate-corrected (model3) RCS curve to assess the relationship of CDAI and vitamin intake with the incidence of COPD. In addition, we used multivariate linear regression models to assess the potential relationship between CDAI and inflammatory factors. Mediator analysis was also used to explore whether inflammatory factors mediated the association between CDAI and COPD prevalence. Interaction and subgroup analyses were conducted based on all covariates with the aim of exploring the stability of the results. Finally, sensitivity analysis was used to check the reliability of the results (1). We removed all data that was missing (2). BMI and PIR were shifted to continuous variables for adjustment (3). Adjustments were made for inflammation (c-reaction protein), liver function (aspartate amino transferase, alanine amino transferase, γ-glutamyl transpeptadase, alkaline phosphatase), healthy eating index (HEI) and total energy intake to see if these indicators affected the relationship between CDAI and COPD (4). RCS curves were plotted for the above sensitivities. Version R4.21 was used for all analyzes in this study.

## 3 Results

### 3.1 Baseline data characteristics

There were 8257 participants in this study (60.0% aged 40-60 years and 40.0% aged > 60 years; female: male ratio was 1:0.97), with 772 COPD participants and 7,485 non-COPD participants. The COPD group had a significantly higher proportion of > 60 years, female, non-Hispanic white, former smokers, current smokers, former drinkers, CKD, and cardiovascular disease than the non-copd group (**Table 1**). In addition, CDAI levels were lower in the COPD and higher age groups, while there were no significant differences in CDAI levels across sex groups (**Supplementary Table S1**).

**Table 1:** Baseline information, weighted.

Characteristic	Overall	Participants without COPD	Participants with COPD	p
N	8257	7485	772	
**Age, n (%)**				<0.001
40-60 years	4004 (60.0)	3739 (61.5)	265 (46.5)	
>60 years	4253 (40.0)	3746 (38.5)	507 (53.5)	
**Sex, n (%)**				0.002
Male	4146 (49.3)	3679 (48.3)	467 (58.3)	
Female	4111 (50.7)	3806 (51.7)	305 (41.7)	
**Race, n(%)**				<0.001
Mexican America	1084 (5.7)	1048 (6.2)	36 (1.2)	
Other races	1341 (9.7)	1254 (10.0)	87 (7.0)	
Non-Hispanic White	4061 (74.3)	3545 (73.2)	516 (84.1)	
Non-Hispanic Black	1771 (10.3)	1638 (10.6)	133 (7.7)	
**Marriage, n(%)**				0.299
Married/living with partner	5088 (67.1)	4632 (67.2)	456 (66.5)	
Widowed/divorced/separated	2543 (25.3)	2270 (25.1)	273 (27.7)	
Never married	626 (7.6)	583 (7.8)	43 (5.8)	
**Education, n (%)**				0.207
<High school	2456 (19.1)	2214 (18.7)	242 (22.5)	
High school	1965 (25.0)	1780 (25.0)	185 (24.5)	
>High school	3836 (56.0)	3491 (56.3)	345 (53.0)	
**Poverty-to-income ratio, n (%)**				0.085
≤1	1362 (10.3)	1214 (10.1)	148 (13.0)	
1-3	3798 (36.8)	3435 (36.5)	363 (39.5)	
>3	3097 (52.9)	2836 (53.4)	261 (47.5)	
**Body mass index, n (%)**				0.384
≤25 kg/m^2^	1987 (25.8)	1778 (25.7)	209 (26.9)	
25-30 kg/m^2^	2990 (36.4)	2709 (36.1)	281 (39.0)	
>30 kg/m^2^	3280 (37.8)	2998 (38.2)	282 (34.1)	
**Smoking status, n (%)**				<0.001
Never smoking	4135 (50.2)	4002 (53.5)	133 (19.2)	
Former smoking	2662 (32.2)	2284 (30.5)	378 (47.8)	
Currently smoking	1460 (17.7)	1199 (16.0)	261 (33.0)	
Alcohol status, n (%)				
Never drinking	1196 (11.5)	1143 (12.1)	53 (6.1)	<0.001
Former drinking	2023 (20.1)	1766 (19.2)	257 (28.5)	
Mild to moderate drinking	4000 (54.7)	3624 (54.9)	376 (52.5)	
Heavy drinking	1038 (13.7)	952 (13.8)	86 (12.9)	
**Hypertension, n (%)**				0.527
No	2519 (35.2)	2309 (35.4)	210 (33.6)	
Yes	5738 (64.8)	5176 (64.6)	562 (66.4)	
**Diabetes, n (%)**				0.326
No	6026 (79.9)	5490 (80.2)	536 (77.9)	
Yes	2231 (20.1)	1995 (19.8)	236 (22.1)	
**Hyperlipidemia, n (%)**				0.728
No	1626 (19.0)	1482 (18.9)	144 (19.7)	
Yes	6631 (81.0)	6003 (81.1)	628 (80.3)	
**Chronic kidney disease, n (%)**				0.04
No	6040 (80.0)	5597 (80.4)	543 (76.8)	
Yes	2117 (20.0)	1888 (19.6)	229 (23.2)	
**Cardiovascular disease, n (%)**				<0.001
No	6822 (86.0)	6267 (86.8)	555 (78.2)	
Yes	1435 (14.0)	1218 (13.2)	217 (21.8)	

COPD, chronic obstructive pulmonary disease.

### 3.2 Dietary antioxidant intake and CDAI in relation to the incidence of COPD

**Figure 1:**
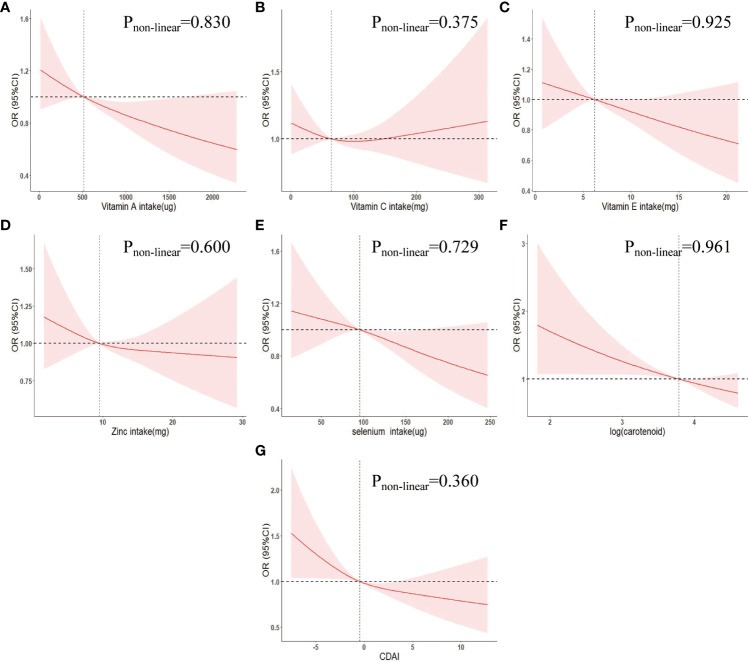
Weighted restricted cubic spline curve describing the dose-response relationship between indicators of dietary antioxidant levels and COPD incidence. **(A)** Vitamin A intake; **(B)** Vitamin C intake; **(C)** Vitamin E intake; **(D)** Zinc intake; **(E)** Selenium intake; **(F)** log(carotenoid intake); **(G)** CDAI. Adjusted for age, gender, race, marriage, education, poverty-to-income ratio, body mass index, smoking status, alcohol status, hypertension, diabetes, hyperlipidemia, chronic kidney disease, cardiovascular disease. OR, odds ratio; 95% CI, 95% confidence interval. CDAI, composite dietary antioxidant index.

In the multivariate adjusted logistic regression model (model 3) of **Table 2**, we found that compared to Q1 intake, log (carotenoid intake) Q2 (OR (95% CI) = 0.67 (0.50-0.91)), Q3 (OR (95% CI) = 0.66 (0.49-0.90)), Q4 (OR (95% CI) = 0.74 (0.54-1.01)) intake all reduced the risk of COPD, and there was an overall decreasing trend in risk with increasing intake (p for trend = 0.052). Multivariate adjusted RCS curves showed a linear decrease in the risk of COPD with intake of most dietary antioxidants (**Figures 1A, C, D–F**), but there was a V-shaped association between vitamin E and COPD (**Figure 1B**).

**Table 2:** Results of a multiple logistic regression analysis of the correlation between antioxidant indicators and COPD, weighted.

Variable	OR (95%CI)		
	Model1^a^	Model2^b^	Model3^c^
Vitamin A intake			
Q1	1.00 (Ref.)	1.00 (Ref.)	1.00 (Ref.)
Q2	0.72 (0.52,0.98)	0.64 (0.46,0.88)	0.70 (0.50,0.96)
Q3	0.89 (0.69,1.17)	0.75 (0.57,0.99)	0.87 (0.64,1.20)
Q4	0.73 (0.51,1.04)	0.58 (0.40,0.83)	0.72 (0.49,1.04)
P for trend	0.189	0.010	0.191
Vitamin C intake			
Q1	1.00 (Ref.)	1.00 (Ref.)	1.00 (Ref.)
Q2	0.71 (0.55,0.92)	0.68 (0.52,0.89)	0.84 (0.61,1.14)
Q3	0.70 (0.53,0.92)	0.65 (0.49,0.88)	0.81 (0.60,1.10)
Q4	0.78 (0.57,1.06)	0.73 (0.54,1.00)	0.97 (0.68,1.39)
P for trend	0.136	0.070	0.824
Vitamin E intake			
Q1	1.00 (Ref.)	1.00 (Ref.)	1.00 (Ref.)
Q2	0.85 (0.60,1.20)	0.82 (0.57,1.17)	0.93 (0.62,1.38)
Q3	0.98 (0.71,1.36)	0.90 (0.64,1.26)	1.01 (0.72,1.42)
Q4	0.67 (0.47,0.95)	0.59 (0.40,0.87)	0.70 (0.46,1.05)
P for trend	0.044	0.011	0.085
Zinc intake			
Q1	1.00 (Ref.)	1.00 (Ref.)	1.00 (Ref.)
Q2	0.97 (0.69,1.36)	0.92 (0.66,1.29)	0.93 (0.67,1.29)
Q3	0.88 (0.61,1.26)	0.81 (0.56,1.17)	0.87 (0.59,1.27)
Q4	1.01 (0.76,1.34)	0.88 (0.65,1.20)	0.94 (0.69,1.28)
P for trend	0.943	0.389	0.697
Selenium intake			
Q1	1.00 (Ref.)	1.00 (Ref.)	1.00 (Ref.)
Q2	0.99 (0.75,1.33)	0.92 (0.69,1.23)	0.93 (0.71,1.23)
Q3	1.07 (0.78,1.48)	0.97 (0.71,1.33)	1.04 (0.75,1.43)
Q4	0.80 (0.60,1.07)	0.72 (0.50,1.03)	0.76 (0.54,1.07)
P for trend	0.207	0.103	0.190
log (Carotenoid intake)			
Q1	1.00 (Ref.)	1.00 (Ref.)	1.00 (Ref.)
Q2	0.64 (0.47,0.87)	0.64 (0.47,0.87)	0.67 (0.50,0.91)
Q3	0.61 (0.45,0.81)	0.59 (0.44,0.80)	0.66 (0.49,0.90)
Q4	0.62 (0.46,0.84)	0.61 (0.45,0.83)	0.74 (0.54,1.01)
P for trend	0.002	0.002	0.052
CDAI			
Q1	1.00 (Ref.)	1.00 (Ref.)	1.00 (Ref.)
Q2	0.67 (0.51,0.88)	0.63 (0.49,0.82)	0.71 (0.53,0.95)
Q3	0.77 (0.56,1.06)	0.72 (0.52,0.99)	0.84 (0.58,1.20)
Q4	0.51 (0.36,0.70)	0.49 (0.35,0.69)	0.61 (0.44,0.83)
P for trend	<0.001	<0.001	0.018

COPD, chronic obstructive pulmonary disease; CDAI, composite dietary antioxidant index;

Vitamin A intake: Q1 (≤ 320.5ug/day), Q2 (320.5 to 513.5ug/day), Q3 (513.5 to 769.5ug/day), Q4 (> 769.5ug/day); Vitamin C intake: Q1 (≤ 31.7mg/day), Q2 (31.7 to 64.3 mg/day), Q3 (64.3 to 110.8mg/day), Q4 (> 110.8mg/day); Vitamin E intake: Q1 (≤ 4.245mg/day), Q2 (4.245 to 6.18mg/day), Q3 (6.18 to 8.715mg/day), Q4 (> 8.715mg/day); Zinc intake: Q1 (≤ 7.005mg/day), Q2 (7.005 to 9.54mg/day), Q3 (9.54 to 12.88mg/day), Q4 (> 12.88mg/day); Selenium intake: Q1 (≤ 70.15ug/day), Q2 (70.15 to 95.6ug/day), Q3 (95.6 to 123.5ug/day), Q4 (> 123.5ug/day); log (Carotenoid intake): Q1 (≤ 3.468), Q2 (3.468 to 3.783), Q3 (3.783 to 4.058), Q4 (> 4.058); CDAI: Q1 (≤ -2.512), Q2 (-2.512 to -0.471), Q3 (-0.471 to 1.878), Q4 (> 1.878);

a Model 1: adjusted for no covariates;

b Model 2: adjusted for basic characteristics (age, sex, and race);

c Model 3: adjusted for all covariates (age, gender, race, marriage, education, poverty-to-income ratio, body mass index, smoking status, alcohol status, hypertension, diabetes, hyperlipidemia, chronic kidney disease, cardiovascular disease).

In addition, using CDAI Q1 levels as a reference, Q2 (OR (95% CI) = 0.71 (0.53,0.95)), Q3 (OR (95% CI) = 0.84 (0.58-1.20)), Q4 (OR (95% CI) = 0.61 (0.44-0.83)) were associated with a decreased risk of COPD and an overall decreasing trend in COPD risk with increasing CDAI (P for trend = 0.018) (**Table 2**). The RCS curve showed a negative linear correlation between CDAI and COPD risk (Pnon-linear = 0.360) (**Figure 1G**).

### 3.3 Association between CDAI and inflammatory

A multiple linear regression model was used to investigate the relationship between CDAI and inflammation (**Supplementary Table S2**). The results showed that CDAI levels were negatively correlated with white blood cells (β = -0.028, 95% CI -0.045, -0.010, P = 0.002), alkaline phosphatase (β = -0.386, 95% CI -0.552, -0.220, P< 0.001), and c-reactive protein (β = -0.009, 95% CI -0.015, -0.004, P< 0.001), but not with neutrophils.

### 3.4 Mediation by inflammatory factors

The effect of inflammatory factors in the relationship between CDAI and COPD prevalence was explored through mediation analysis. As shown in **Table 3**, alkaline phosphatase and c-reactive protein partially mediated the association between CDAI and COPD prevalence, with a mediation ratio of 6.4% (P< 0.01) and 4.68% (P = 0.04), respectively. White blood cells and neutrophils had no significant mediating effect (P > 0.05).

**Table 3:** The mediating effects of the relationship between CDAI and COPD risk.

Inflammatory factors	Indirect effects	Direct effects	Total effects	Mediated proportion (%)	P-value
	β (95%CI)	β (95%CI)	β (95%CI)		
White blood cells	-8.43e-07 (-6.27e-05, 0.00)	-1.81e-03 (-3.73e-03, 0.00)	-1.81e-03 (-3.73e-03, 0.00)	0.05	0.98
Neutrophil cells	-3.82e-07 (-5.02e-05, 0.00)	-1.75e-03 (-3.86e-03, 0.00)	-1.75e-03 (-3.86e-03, 0.00)	0.02	1
Alkaline phosphatase	-1.28e-04 (-2.37e-04, 0.00)	-1.85e-03 (-3.95e-03, 0.00)	-2.00e-03 (-4.11e-03, 0.00)	6.4	<0.01
C-reactive protein	-8.37e-05 (-1.70e-04, 0.00)	-1.71e-03 (-3.61e-03, 0.00)	-1.79e-03 (-3.70e-03, 0.00)	4.68	0.04

COPD, chronic obstructive pulmonary disease; CDAI, composite dietary antioxidant index.

### 3.5 Subgroup analysis

**Figure 2:**
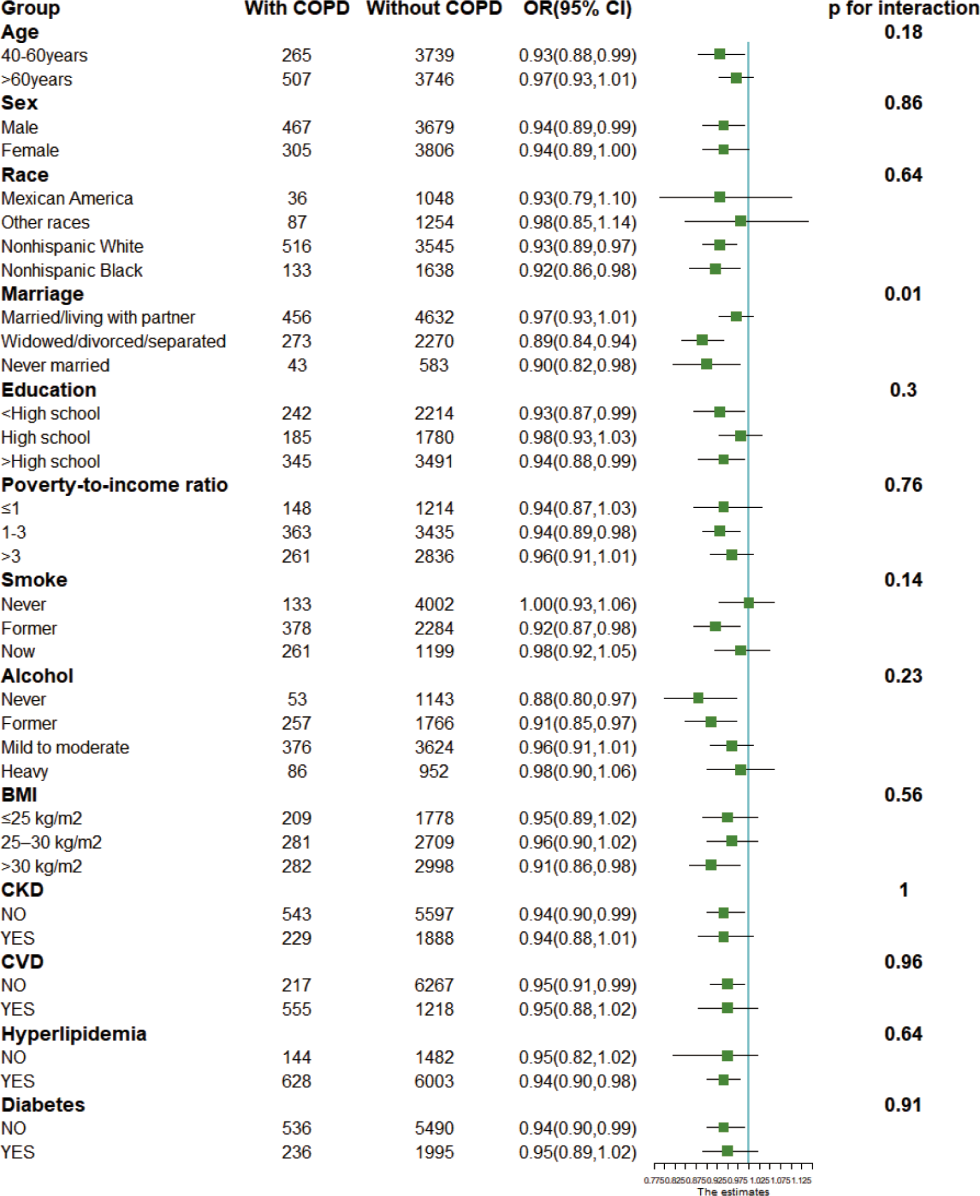
Forest plots for subgroup analysis. Subgroup analysis was stratified by age, sex, race, marriage, education, poverty index, body mass index, drinking status, smoking status, and disease status. BMI, body mass index; CKD, chronic kidney disease; CVD, cardiovascular disease. OR, odds ratio; 95% CI, 95% confidence interval.

We performed subgroup analyzes and interactions, and visualized the OR and 95% CI of studied subgroups by drawing forest plots (**Figure 2**). The results showed a stable negative association between CDAI and the incidence of COPD in most populations (p for interaction > 0.1). Also, CDAI had an overall protective effect against COPD in all subgroups (OR ≤ 1).

### 3.6 Sensitivity analysis

We conducted several sensitivity analyzes. First, when excluding participants with missing covariates (model 2, **Supplementary Table S3**) or adjusting for BMI and IFP by switching to continuous variables (model 3, **Supplementary Table S3**), the results were largely unchanged. Secondly, when adjusting for the inflammatory index (model 4, **Supplementary Table S3**), the liver function index (model 5, **Supplementary Table S3**), HEI (model 6, **Supplementary Table S3**) and total energy intake (model 7, **Supplementary Table S3**), respectively, the results remained unchanged significantly. Thirdly, the RCS curves were plotted according to the sensitivity analysis described above (**Supplementary Figure S2**) and the graphical curves were approximately the same. Therefore, our conclusions have strong stability.

## 4 Discussion

In this cross-sectional study, we used NHANES data from 2007-2012 to investigate the association between CDAI and dietary antioxidant intake with COPD risk in US adults aged ≥ 40 years. The weighted prevalence of COPD was 9.8% in this study. In multivariate logistic regression analysis, higher carotenoid intake and CDAI levels were found to be independently associated with lower COPD prevalence. Also, RCS curves showed a negative linear relationship between log (carotenoids) and CDAI with COPD. Therefore, carotenoid supplementation and dietary patterns high in antioxidants appear to be important for COPD prevention in patients aged ≥ 40 years.

A cross-sectional study in the US population showed that vitamin C, vitamin E and total carotene were all positively associated with forced expiratory volume in 1 s (FEV_1_), with total carotene having the largest effect. Meanwhile the combined effect of dietary intake of these antioxidants was higher than when any single component was considered alone (12). This is similar to our findings and a diet high in carotenoids or antioxidants may be able to reduce the incidence of COPD by improving lung function. In addition, a 3-year randomized controlled study comprising 120 COPD patients suggested that high antioxidant food intake may be associated with improved lung function and that dietary interventions could be considered in the management of COPD patients (13). We therefore sought to use the CDAI to assess whether high antioxidant food intake could play the same role in the prevention of COPD.

Carotenoids are fat-soluble vitamins with antioxidant properties, mainly found in yellow or orange fruits and vegetables. The current experimental results validate the potential efficacy of carotenoids in the treatment of COPD (14). In a mouse model of nicotine-induced lung cancer and emphysema, β-cryptoxanthin, an oxygenated carotenoid, can restore nicotine-suppressed lung SIRT1, p53, and RAR-β expression, and improve survival. It also reduced lung IL-6 and AKT phosphorylation levels and improved emphysema (15). Lycopene is rich in red fruits and vegetables. In a mouse emphysema model induced by cigarette smoke (CS), lycopene can reduce leukocytes in bronchoalveolar lavage fluid and attenuate emphysema. In this experiment, lycopene also can increased the activity of antioxidant enzymes (catalase, superoxide dismutase and glutathione) and reduced redox processes, lipid peroxidation and DNA damage (16). Astaxanthin is a xanthophyll carotenoid mostly found in marine organisms. In a CS-induced COPD mouse model, astaxanthin intake activated the Nrf2-ARE signaling pathway to inhibit inflammatory cell infiltration and significantly improved emphysema (17). However, most of the studies were CS-induced models, and the role of carotenoids in other COPD models needs to be further explored. Epidemiologically, the role of carotenoid intake on lung function is controversial. Previous studies have shown a positive association between total carotenoid intake and lung function, but some studies have shown no association (12, 18, 19). In addition, large cross-sectional studies of dietary carotenoid intake and COPD incidence are lacking. In the present study, we found that increased carotenoid intake reduced the incidence of COPD.

CDAI was developed to explore the overall effects of dietary antioxidants on the human body. Previous studies have found that high CDAI is associated with a reduced risk of diseases such as cancer, CKD and depression (7–9, 20). Notably, participants in the higher age group had a lower CDAI, and these results may be due to the different appetite, and eating habits of individuals of different ages (21). In addition, one study (22) has shown that CDAI is negatively associated with IL-1β and TNF-α, and in our study CDAI was also found to be negatively associated with inflammatory factors such as white blood cell, alkaline phosphatase and c-reactive protein. Age, gender, smoking, income, and race are important factors in the progression of COPD (23). A stable association of CDAI with COPD remained after adjustment for these indicators, suggesting that antioxidant diet may be used as primary prevention of COPD, but the mechanism for this potential association is unclear. On the one hand, circulating antioxidants may act directly on the lungs. On the other hand, dietary antioxidants reduce COPD risk by mediating alkaline phosphatase, c-reactive protein and other inflammatory factors to reduce inflammation in the lung. The present study suggests that CDAI may be a comprehensive assessment of COPD risk in healthy individuals, and more research is needed to explore the role of CDAI in the future.

There are several strengths: First, this study explored the independent association of six dietary antioxidants with COPD and first suggested the feasibility of CDAI to detect COPD risk. Second, there was a stable relationship between CDAI and COPD in different subgroups. Third, several sensitivity analyzes were performed in this study to further validate the reliability of the results.

Several weaknesses exist: First, dietary antioxidant intake was defined as the mean of two 24-h recall interviews, which were subject to recall bias. Secondly, unknown confounders could not be avoided. Thirdly, there may be selection bias as many subjects were excluded from the study due to missing or unavailable data. Furthermore, based on the NHANES database characteristics, we considered criteria such as self-report as indicators of a diagnosis of COPD, which may be subject to reporting bias. Finally, this study was cross-sectional and it was not possible to infer a causal relationship between high dietary antioxidant intake and COPD.

## 5 Conclusion

In adults aged ≥ 40 years, a diet high in carotenoids or antioxidants may be a primary preventive measure for COPD, and CDAI may be used clinically as a predictor of COPD risk. However, further research is needed to determine whether antioxidant factors can reduce the risk of complications and mortality in COPD patients.

## Data availability statement

Publicly available datasets were analyzed in this study. This data can be found here: The dataset used for statistical analysis in this study is available on the NHANES website: https://www.cdc.gov/nchs/nhanes/index.htm.

## Ethics statement

The studies involving humans were approved by Institutional Review Board of the National Center for Health Statistics. The studies were conducted in accordance with the local legislation and institutional requirements. The participants provided their written informed consent to participate in this study.

## Author contributions

ZL: Formal analysis, Methodology, Software, Writing – original draft, Writing – review & editing. JL: Conceptualization, Data curation, Investigation, Writing – original draft, Writing – review & editing. TC: Investigation, Software, Writing – review & editing. XZ: Formal analysis, Writing – review & editing. QC: Investigation, Writing – review & editing. LX: Investigation, Writing – review & editing. ZP: Project administration, Supervision, Validation, Writing – review & editing. HZ: Funding acquisition, Project administration, Supervision, Visualization, Writing – review & editing.
