# Supplementary material for: Association between dietary antioxidant levels and chronic obstructive pulmonary disease: a mediation analysis of inflammatory factors
Source: Front Immunol. 2024 Jan 8;14:1310399. doi: 10.3389/fimmu.2023.1310399 (PMC10800866; doi:10.3389/fimmu.2023.1310399)
Supplement: Supplementary file 1 [file DataSheet_1.docx]

Supplementary Material

1. **Supplementary Figures**


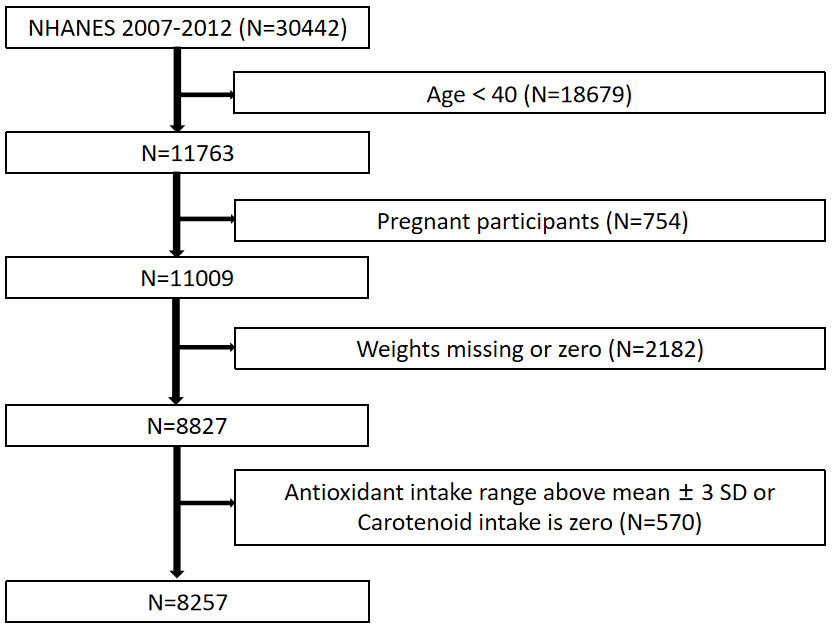


**Supplemental Figure 1.** Flowchart of participant screening based on NHANES database from 2007-2012.


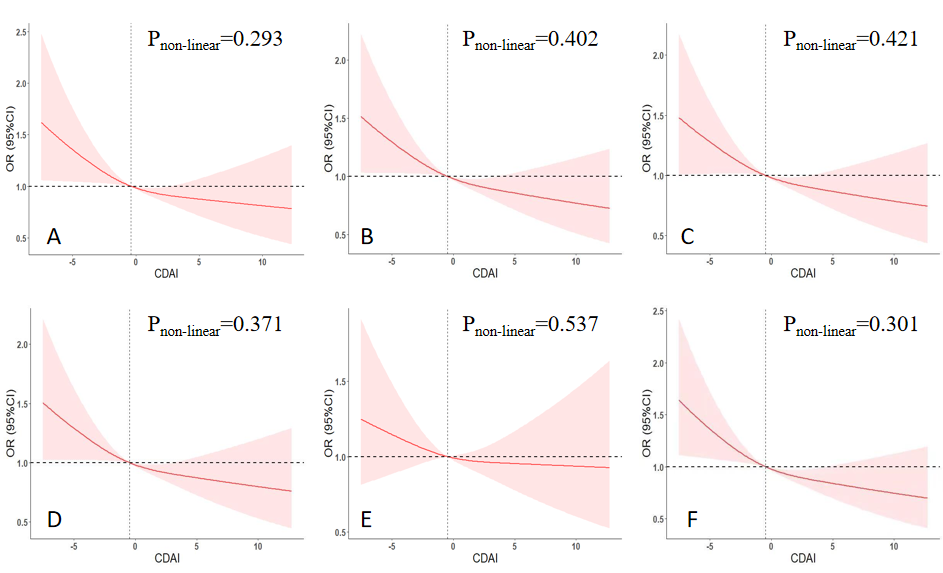


**Supplemental Figure 2. Dose-response relationship between CDAI levels and COPD incidence after further adjustment for data composition, biomarkers and dietary factors.**

All covariates were adjusted in these models.

A, Remove all missing data;

B, BMI and PIR were shifted to continuous variables for adjustment.;

C, Further adjustments to CRP;

D, Further adjustments to AST, ALT, GGT, ALP;

E, Further adjustments to total energy intake;

F, Further adjustments to HEI.

# 2. Supplementary Tables

**Supplemental Table 1.** Mean CDAI levels by age, sex and COPD group.

|  | 40-60 years | >60 years | Male | Female | Participants without COPD | Participants with COPD |
| --- | --- | --- | --- | --- | --- | --- |
| CDAI | 0.68±3.45 | 0.03±3.23 | 0.40±3.15 | 0.44±3.58 | 0.60±3.53 | 0.08±3.60 |
| P value | ＜0.001 | | 0.719 | | 0.013 | |

**Supplemental Table 2.** Multivariate linear regression of CDAI levels and inflammatory factors.

|  | β | 95%CI | P-value |
| --- | --- | --- | --- |
| White blood cells (1000 cells/μL) | -0.028 | -0.045, -0.010 | 0.002 |
| Neutrophils (％) | 0.001 | -0.062, 0.065 | 0.963 |
| Alkaline phosphatase (u/L) | -0.386 | -0.552, -0.220 | <0.001 |
| C-reactive protein (mg/dl) | -0.009 | -0.015, -0.004 | <0.001 |

CDAI, composite dietary antioxidant index.

**Supplemental Table 3.** Relationship between CDAI levels and COPD incidence after further adjustment for data composition, biomarkers and dietary factors.

|  | OR (95% CI) | | | | P for trend |
| --- | --- | --- | --- | --- | --- |
|  | Q1 | Q2 | Q3 | Q4 |  |
| Model 1 | 1.00 (Ref.) | 0.71 (0.53,0.95) | 0.84 (0.58,1.20) | 0.61 (0.44,0.83) | 0.018 |
| Model 2 | 1.00 (Ref.) | 0.67 (0.49,0.92) | 0.94 (0.62,1.43) | 0.59 (0.41,0.84) | 0.041 |
| Model 3 | 1.00 (Ref.) | 0.71 (0.53,0.96) | 0.84 (0.59,1.21) | 0.60 (0.44,0.84) | 0.020 |
| Model 4 | 1.00 (Ref.) | 0.71 (0.53,0.95) | 0.84 (0.59,1.22) | 0.61 (0.45,0.84) | 0.020 |
| Model 5 | 1.00 (Ref.) | 0.71 (0.52,0.96) | 0.85 (0.59,1.23) | 0.62 (0.45,0.85) | 0.025 |
| Model 6 | 1.00 (Ref.) | 0.68 (0.50,0.92) | 0.80 (0.55,1.14) | 0.56 (0.40,0.80) | 0.010 |
| Model 7 | 1.00 (Ref.) | 0.74 (0.56,0.99) | 0.92 (0.62,1.35) | 0.70 (0.50,0.99) | 0.187 |

Model 1: adjusted for all covariates (age, gender, race, marriage, education, poverty-to-income ratio, body mass index, smoking status, alcohol status, hypertension, diabetes, hyperlipidemia, chronic kidney disease, cardiovascular disease).

Model 2: Model 1 + remove all missing data (Q1 (≤ -2.417), Q2 (-2.417 to -0.379), Q3 (-0.379 to 1.988), Q4 (> 15.948);

Model 3: Model 1 + BMI and PIR were shifted to continuous variables for adjustment.

Model 4: Model 1 + CRP

Model 5: Model 1 + AST, ALT, GGT, ALP

Model 6: Model 1 + HEI

Model 7: Model 1 + total energy intake
